# Yield and quality attributes of faba bean inbred lines grown under marginal environmental conditions of Sudan

**DOI:** 10.1002/fsn3.245

**Published:** 2015-05-19

**Authors:** Seif Gasim, Solafa A.A. Hamad, Awadalla Abdelmula, Isam A. Mohamed Ahmed

**Affiliations:** ^1^ Department of AgronomyFaculty of AgricultureUniversity of KhartoumShambatSudan; ^2^ Food Research CenterMinistry of Science and CommunicationsKhartoumShambatSudan; ^3^ Department of Food Science and TechnologyFaculty of AgricultureUniversity of KhartoumShambat13314Sudan

**Keywords:** Faba bean, HJ‐biplot, protein, protein fractions, seed yield

## Abstract

Faba beans (*Vicia faba* L.) represent an essential source of food protein for many people in Sudan, especially those who cannot afford to buy animal meat. The demand for faba bean seeds is greatly increased in recent years, and consequently its production area was extended southward where the climate is marginally suitable. Therefore, this study was aimed to evaluate seed yield and nutritional quality of five faba bean inbred lines grown under marginal environmental conditions of Sudan. The inbred lines have considerable (*P *≤* *0.05) variability in yield and yield components, and seed chemical composition. The mean carbohydrate content was very high (501.1 g kg^−1^) and negatively correlated with seed yield, whereas the average protein content was relatively high (253.1 g kg^−1^) and positively correlated with seed yield. Globulin was the significant fraction (613.5 g kg^−1^protein) followed by albumin (200.2 g kg^−1^protein). Biplot analysis indicates that inbred lines Hudeiba/93‐S5 and Ed‐damar‐S5 outscore other lines in terms of seed yield and nutritional quality. This study demonstrates that Hudeiba/93‐S5 and Ed‐damar‐S5 are useful candidates in faba bean breeding program to terminate the protein deficiency malnutrition and provide healthy and nutritious meal for people living in subtropical areas.

## Introduction

Faba bean (*Vicia faba* L.) is one of the most important legume crops used as food for human consumption in developing countries and as animal feed in advanced countries. It is the fourth important pulse crop in the world after dry bean, dry peas, and chickpeas (Kumari and Van Leur 2011). Its value as a food and feed crop lies in its high lysine‐rich protein, vitamins, minerals, and carbohydrates (Crepon et al. 2010), which make it one of the best solutions to the malnutrition, particularly in developing countries in Africa and parts of Asia and Latin America (Haciseferogullari et al. 2003). The crop is also used as an excellent component of crop rotations, something that has been very much neglected in modern cropping, at a time when there is an urgent need to minimize the impact of chemical fertilizers on the environment, reduce emissions of undesirable grasses (Kopke and Nemecek 2010), and reduce the production cost of the following crops. Faba bean is a winter crop that grows better under cool and moist conditions, whereas, hot and dry weather is unfavorable and could lead to the decrease in the seed yield and quality (Flores et al. 2012). This plant is considered to be the least drought‐resistant legume crop, however, most of the breeding programs are directed toward improving the drought resistance of this crop and accordingly cultivars with high water use efficiency have recently been developed at ICARDA (Subash and Priya 2012). Owing to its numerous uses, great nutritional quality and ability to grow over a broad range of climatic and soil conditions, faba bean is appropriate for sustainable agriculture in many marginal areas (Nadal et al. 2003), and the crop has gained greater global attention in recent years.

In Sudan, faba bean is one of the primary grown and consumed legume crops. It constitutes to the primary human nutrition, supplying high‐quality proteins essential for a balanced diet for the daily breakfast and dinner of the millions of people who cannot afford meat as a source of protein in both rural and urban area (Osman et al. 2014). In many parts of Sudan, faba beans are served in several types of dishes such as stewed faba bean (Fuel Musalah), deep fried cotyledon paste with some vegetables and spices (Taamia or Falafel), and faba bean soup with bread and cheese whey (Fata). Additionally, the crop is an imperative source of income for the farmers in the country (Salih and Mohamed 1992). However, the demand for this nutritious legume crop is growing, fuelled by rapid population growth in the country, which led to an enormous gap between the supply and demand. In Sudan, faba bean is traditionally cultivated in the banks of the Nile River north of latitude 18.50°N in the Northern and Nile States, where temperature is moderately cooler and winter longer (Salih and Mohamed 1992). However, to meet the ever‐increasing demand for faba bean in Sudan, its production was extended into the warmer areas at latitudes lower than 15°N, where the climate is marginally suitable. In these areas, faba bean yield is far below the potential (Gasim et al. 2013), mainly because of the biological limitations of the traditional cultivars and poor management practices as well as the effect of abiotic (especially temperature) and/or biotic (diseases and pests) stresses. Improving seed yield and quality of faba bean under stress conditions are important priorities to meet the increasing demand and feed a growing population. Thus, the breeding objectives for this crop have always been and still are to improve the resistances to drought, heat, diseases, and pests, as well as to enhance the grain yield and quality. However, evidence on the yield and nutritional quality of newly developed faba bean inbred lines under marginal environment of Sudan is scarce. Detailed information about the productivity and seeds quality of faba beans inbred lines grown in nontraditional areas of Sudan will enhance our knowledge and contribute to the food security and income of the growing population in semiarid areas. Therefore, the primary aim of the present study was to investigate the seed yield and quality of five faba bean inbred lines grown under the marginal environmental conditions of Sudan.

## Materials and Methods

### Planting material and field trials

**Figure 1:**
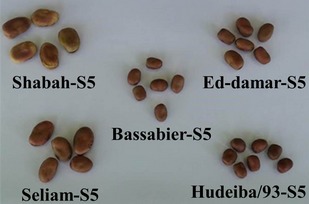
The seeds of five faba beans inbred lines grown in the marginal environment in Sudan. The seeds display different color, shape, and size depending on the genotype variations. The seed size of inbred lines Shabah‐S5 and Seliam‐S5 is bigger than that of the other three inbred lines. The parents of these two inbred lines initially originate from the northern part of Sudan, where the environmental condition is suitable for faba bean cultivation. In Sudan, the parents of the inbred lines Shabah‐S5 and Seliam‐S5 have high marketability, and consumer preference compared to those of the three other genotypes. On the other hand, the parents of the inbred lines Hudeiba/93‐S5 and Ed‐damar‐S5 have high yield and farmer's preference in the country.

The planting materials used consisted of five faba bean lines produced by single plant selection under insect‐proof cages from their corresponding known cultivars, namely Bassabier, Seliam, Hudeiba/93, Shabah, and Ed‐damar. These inbred lines differ mainly in seed size, seed coat color (Fig. 1), plant height, and susceptibility to biotic stresses. The material was planted at Shambat Farm (latitude 15˚39°N, longitude 32°31 E, and altitude 380 meters above the sea level) of the Faculty of Agriculture, University of Khartoum, Sudan for two consecutive growing seasons (2011/2012, 2012/2013), in a randomized complete block design with three replications. The gross plot size was 9 m^2^ consisting of eight ridges, 70 cm apart. The spacing between holes was 20 cm. The lines were sown during the second week of November in both seasons. The experimental plots were irrigated at an average interval of 12 days and hand weeded after three weeks and then whenever it was necessary using hand hoeing. The average mean temperature during the growing season was 28°C. The soil of the Experimental Farm is heavy clay with alkaline pH (8.0).

### Yield parameters

The traits of the inbred lines were examined in terms of number of pods/plant, seeds/pod, and seeds/plant, 100‐seed weight (g), and yield (kg ha^−1^) as well as seed quality.

### Chemical composition

The contents of moisture (950.46B), ash (920.153), fiber (962.09), lipids (960.39), crude protein (981.10), and carbohydrate of the faba beans seed samples (*n* = 3) were determined following the official methods of analysis (AOAC 2003). Carbohydrate content was determined by subtracting the content of moisture, protein, ash, fiber, and fat from 100.

### Protein fractionation

The sequential extraction of all proteins was carried out according to the method described by Guo and Yao (2006). Three independent samples of faba bean seeds of each inbred line were grinded and sieved through a 0.15 mm square aperture sieve‐mesh and then defatted with hexane. The defatted flour (defatted flour/solvent ratio 1:10 w/v) was sequentially extracted with each of distilled water (albumin), 1.0 mol L^−1^ NaCl (globulin), 70% (v/v) aqueous ethanol (prolamin), and 0.1 mol L^−1^ NaOH (glutelin) for 2 h each at 25°C under continuous stirring. Following each extraction procedure, the mixture was centrifuged at 6000 ×  g at 4°C for 30 min. In order to extract most of the protein, each extraction step was performed twice. The supernatants containing desired protein fractions were frozen, concentrated, and the nitrogen content of each of these fractions was determined by the micro‐Kjeldahl method (AOAC 2003). The residue left after extraction was also analyzed for nitrogen content.

### Statistical analysis

The agronomical and biochemical figures of three independent samples of each inbred line were statistically analyzed using PLABSTAT software (Utz 1997). Correlation coefficients for the mean values of yield and physicochenical quality traits of five inbred lines were evaluated using Stat View software. Multivariate statistical analysis of the data was performed using HJ‐biplot methods included in the MULTBIPLOT software (Vicente‐Villardón 2010). The HJ‐Biplot method allows the plotting of both inbred lines together with their yield and quality traits for an optimum quality of representation and hence provides easy and fast information about the interrelation of the plotted data. In order to ascribe a set of individuals to a particular group, we performed hierarchical clustering analysis with the Euclidean distance using the principal components scores and the Ward's technique as the process of linkage. Significance was accepted at *P *≤* *0.05 and *P *≤* *0.01.

## Results and Discussion

### Yield and yield components

The results showed different (*P *≤* *0.05) values for yield and yield‐related components in the studied faba bean inbred lines (Table 1). The range values were from 6.9 to 10.1 for number of pods/plant, 1.9 to 2.0 for number of seeds/pod, 12.4 to 22.2 for number of seeds/plant, 43.2 to 61.1 for 100‐seed weight and from 2514.1 to 2712.2 kg ha^−1^ for seed yield. Hudeiba/93‐S5 showed the highest number of pods/plant (10.1), number of seeds/plant (22.2), and yield (2712.2 kg ha^−1^) followed by Ed‐damar‐S5 and then Bassabier‐S5. The highest 100‐seed weights (g) were associated with Shabah‐ S5 (61.1) and Seliam‐S5 (60.7) those produced the low yield compared to other lines. These results indicated the presence of varaibility among the inbred lines for yield and yield componenets, which are similar to those obtained in previous faba bean genotypes by other investigators (Karadavut et al. 2010). This variability could be either due to genetic factors or to both genetic and environmental factors (Flores et al. 2012). The observed significant variability among the faba bean inbred lines could provide a valuable genetic resource for plant breeders to develop high‐yielding faba bean genotypes under the marginal environmental conditions (Gasim et al. 2013).

**Table 1:** Yield and yield components of faba bean lines grown under marginal environmental conditions

Genotype/Parameter	No. of pods/plant	No. of seeds/pod	No. of seeds/plant	100‐seed Weight (g)	Yield (kg ha^−1^)
Bassabier‐S5	9.1	2.3	22.2	43.6	2623.7
Seliam‐S5	6.9	1.9	12.4	60.7	2579.1
Hudeiba/93‐S5	10.1	2.5	23.6	43.2	2712.2
Shabah‐S5	7.6	2.0	14.9	61.1	2614.1
Ed‐damar‐S5	9.8	2.4	22.4	45.7	2706.4
Mean	8.7	2.2	19.9	50.9	2627.1
C.V %	18.8	18.7	18.6	06.1	13.9
LSD (0.05)	2.1	0.1	6.3	0.3	47.2

Values are expressed as means of three independent samples.

### Chemical composition

The chemical composition of the seeds of five faba bean inbred lines differed (*P *≤* *0.05) significantly in all quality parameters (Table 2). Moisture content of the inbred lines was ranged from 78.3 to 79.5 g kg^−1^ similar to the value reported by Salim (2009). Ash content ranged from 32.8 to 42.9 g kg^−1^ which agreed with the values in the range of 38.0–42.0 g kg^−1^ reported for various faba bean seeds (Khalil and Mansour 1995; Vioque et al. 2012; Lizarazo et al. 2014). Our findings are also slightly higher than the range 27.0–35.0 g kg^−1^ reported for many faba bean genotypes (Haciseferogullari et al. 2003). The fiber content of inbred lines in the current study was in the range of 112.3 to 122.4 g kg^−1^, which is higher than those reported previously for various faba bean genotypes (Duc et al. 1999), but lower than those reported by Vioque et al. (2012) for Spanish genotypes and by Lizarazo et al. (2014) for genotypes cultivated in Finland. From nutritional standpoint, high fiber content in faba bean demonstrated the expected beneficial effect of consuming faba beans to improve the food digestibility and bowel movement through the human digestive tract thus reduce the risk of many intestinal and colonic diseases. The variations in fiber content within the inbred lines could be attributed to the genetic differences, harvest/postharvest practices, and the growing environment mainly soil fertility, temperature, and humidity. The lower fat content (9.5–12.5 g kg^−1^) of the present faba beans inbred lines compared to other chemical constituents agreed with the fact that legumes always characterized by their low‐fat content (Vioque et al. 2012). Many previous reports indicated fat content within the range of 7.0–20.0 g kg^−1^ for various faba beans genotypes (Khalil and Mansour 1995; Haciseferogullari et al. 2003; Crepon et al. 2010; Vioque et al. 2012; Lizarazo et al. 2014). The crude protein content of many edible legume seeds is usually more than 200 g kg^−1^ and varies considerably depending on the genotypes (Kapoor et al. 1992). The range value of protein content of the inbred lines was from 189.9 g kg^−1^ for Seliam‐S5 to 314.1 g kg^−1^ for Shabah‐S5, which is comparable to the range reported previously for other faba bean genotypes (Avola et al. 2009; Crepon et al. 2010). The extremely low protein concentration of Seliam‐S5 and Hudeiba/93‐S5 could be attributed to the variation in the genotype makeup, the environmental factors mainly soil fertility and temperature, and the interaction between the genotypes and the growing environment. Enough genetic variability is needed in order to improve the seed protein content of faba beans genotypes (Ouji et al. 2010). Carbohydrate content of the lines ranged from 438.2 g kg^−1^ for Shabah‐S5 to 571.8 g kg^−1^ for Seliam‐S5 with an average of 501.1 g kg^−1^. In addition to the effect of the genotype (Kapoor et al. 1992), environmental factors such as heat stress (temperature), drought stress, soil compaction, pests, and diseases might have impacts on nitrogen availability and thus may affect the seed quality.

**Table 2:** Chemical composition (g kg^−1^) of the seeds of faba beans inbred lines grown under marginal environmental conditions

Genotype	Moisture	Ash	Fiber	Fat	Protein	Carbohydrate
Bassabier‐S5	78.7	32.8	121.8	11.1	286.8	469.0
Seliam‐S5	78.9	37.6	112.3	9.5	189.9	571.8
Hudeiba/93‐S5	78.4	38.0	120.4	12.1	201.8	548.7
Shabah‐S5	79.5	42.9	112.6	12.5	314.1	438.2
Ed‐damar‐S5	78.3	38.2	122.4	10.3	272.9	477.9
Mean	78.7	37.9	117.9	11.1	253.1	501.1
LSD (0.05)	0.6	1.1	1.3	1.0	0.6	2.5

Values are expressed as means of three independent samples.

### Protein fractions

The total seed protein content was fractionated according to their solubility into five different fractions: albumin (water‐soluble), globulin (salt‐soluble), prolamins (alcohol‐soluble), glutelins (soluble in dilute NaOH), and residue (Osborne 1924). Protein fractions revealed significant (*P *≤* *0.05) variability among faba beans inbred lines (Table 3). The major protein fraction was the globulin that ranged from 603.0 to 625.9 g kg^−1^protein with an average of 613.5 g kg^−1^protein, followed by albumin (183.7–219.1 g kg^−1^protein) with a mean of 200.2 g kg^−1^protein. Bassabier‐S5 showed the highest globulin and the lowest albumin contents, whereas Hudeiba/93‐S5 revealed the highest albumin and Ed‐damar‐S5 gave the lowest globulin. These results agreed with those reported previously in many faba bean genotypes in which the storage protein globulin was found to be a significant fraction (Ouji et al. 2010). Globulin as the major protein fraction and albumin as the second fraction were reported in other legumes such as cowpea (Chan and Phillips 1994), peas (Rubio et al. 2014), and lupin (Gulewicz et al. 2008). Within the other factions, glutelin showed slightly higher amount compared to the prolamin and the residue (insoluble proteins). The values of these fractions were ranged from 101.8 to122.1 g kg^−1^protein for glutelin, 33.6 to 42.9 g kg^−1^protein for prolamin, and 18.8 to 68.6 g kg^−1^protein for residue. The results indicated that the content of the protein fractions of the faba beans inbred lines was diverse and showed a complete dependence on genotype as well as of the environmental factors (Hossain and Mortuza 2006). Moreover, maturation stage of the seed depends on environmental factors could also lead to variation in the protein fraction composition. Other factors, such as lipid content, particle size, flour solvent ratio, and extraction temperature, seemed to be critical variables in determining the protein fractions (Barba de la Rosa et al. 1992).

**Table 3:** Protein fractions (g kg^−1^ protein) of the seeds of faba beans inbred lines grown under marginal environmental conditions

Genotype	Albumin	Globulin	Prolamin	Glutelin	Residue
Bassabier‐S5	183.7	625.9	33.6	101.8	68.6
Seliam‐S5	195.1	611.5	42.9	113.1	37.3
Hudeiba/93‐S5	219.1	615.2	36.9	122.1	18.8
Shabah‐S5	203.2	611.9	41.5	102.5	40.7
Ed‐damar‐S5	199.7	603.0	39.0	112.2	36.8
Mean	200.2	613.5	38.8	110.4	40.4
LSD (0.05)	10.0	5.6	1.4	1.8	2.3

Values are expressed as means of three independent samples.

### Correlations among yield and quality attributes

Correlation analysis of faba bean yield with physicochemical quality parameters is an essential way to understand the associations between yield and quality characteristics and their interacted influence on each other, and therefore proper assessment of the overall quality of faba bean inbred lines. The results in Table 4 present the correlation between the yield and the physicochemical quality traits of the seeds of five faba beans inbred lines grown in Khartoum, Sudan. The results showed varied correlations (positive, negative, weak) among most of the parameters. The grain yield, fat, and glutelin have no significant correlation with all physicochemical quality traits of all inbred lines, indicating that breeding for quality attributes might not affect the yield of these inbred lines. The 100‐seed weight showed a significant positive correlation (*p <* *0.05, *r* ^2 ^= 0.90) with carbohydrate content, while it showed highly significant (**p <* *0.01) negative correlation (*r* ^2 ^= −0.97) with fiber content and a significantly (*p <* *0.05) negative correlation (*r* ^2^ = −0.91) with globulin. Moisture content also showed significantly (*p <* *0.05) positive correlation (*r* ^2^ = 0.88) with carbohydrate content. These results demonstrated that breeding for faba bean grains filling quality and marketability would significantly improve the carbohydrate content. Ash, protein, albumin, and globulin indicated significantly (*p <* *0.05) negative correlations with globulin (*r* ^2^ = −0.89), carbohydrate (*r* ^2^ = −0.91), residue (*r* ^2^ = −0.93), and prolamin (*r* ^2^ = −0.91), respectively. As the importance of faba bean as food and feed is mainly due to its high protein contents, the inverse relation between protein and carbohydrate could have an adverse impact on the value of faba beans as food and feed (Duc et al. 2011). Fiber shows significantly (*p <* *0.05) positive correlations with protein (*r* ^2^ = 0.92) and globulin (*r* ^2^ = 0.88), while it indicates highly significant (**p <* *0.01) negative correlation with carbohydrate (*r* ^2^ = −0.96). Interestingly, the positive association between fiber and protein is of considerable importance from a nutritional standpoint, indicating that faba bean seeds with high protein content would have high fiber content and thus considered nutritionally sound and potentially healthy. Overall, these correlations demonstrated that breeding for high‐yielding faba bean lines with high protein and fiber content is genetically feasible. In addition, it pointed out that breeders, who are interested in improving carbohydrate content of the faba bean seeds, should have to consider its negative relationship with protein, fiber, and yield.

**Table 4:** Correlation coefficients among 13 grain yield and physicochemical attributes of five faba bean inbred lines grown under marginal environment

	Yield	100‐Seed weight	Moisture	Ash	Fiber	Fat	Protein	Carbohydrate	Albumin	Globulin	Prolamin	Glutelin
100‐Seed weight	−0.75											
Moisture	−0.74	0.83										
Ash	−0.01	0.63	0.56									
Fiber	0.75	−0.97**	−0.82	−0.60								
Fat	0.27	−0.11	0.35	0.41	0.02							
Protein	0.74	−0.80	−0.69	−0.35	0.92*	0.01						
Carbohydrate	−0.67	0.90*	0.88*	0.66	−0.96**	0.25	−0.91*					
Albumin	0.60	−0.09	−0.17	0.56	−0.03	0.50	−0.07	0.16				
Globulin	0.43	−0.91*	−0.75	−0.89*	0.88*	−0.14	0.66	−0.87	−0.26			
Prolamin	−0.41	0.87	0.49	0.73	−0.83	−0.26	−0.62	0.71	0.19	−0.91*		
Glutelin	0.58	−0.29	−0.62	0.04	0.18	−0.12	0.01	−0.19	0.75	0.11	0.10	
Residue	−0.49	−0.07	0.22	−0.56	0.15	−0.15	0.17	−0.19	−0.93*	0.37	−0.45	−0.85

Significant level: *p <* *0.05; **p <* *0.01.

### HJ‐biplot analysis

**Figure 2:**
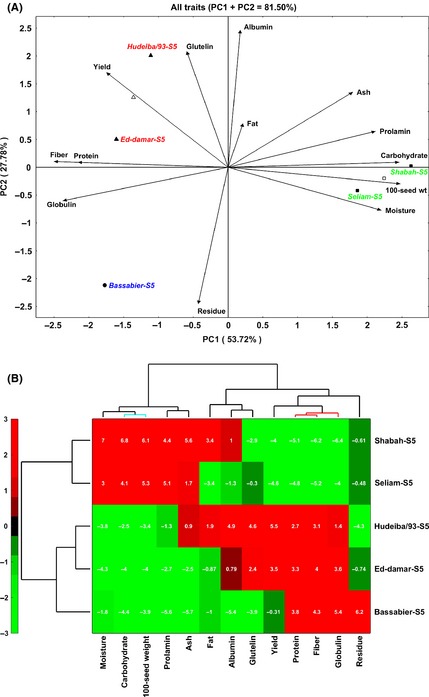
The HJ‐biplot based on principal component analysis for yield and quality attributes of five faba bean inbred lines grown in the marginal environment in Sudan. In the ballots, we used hierarchical clustering analysis with the Euclidean distance using the principal components scores and the Ward's technique as the process of linkage.The biplot shows the interrelations between the yield and quality traits of the inbred lines (A). Bidimensional clustering analysis indicating the relationships between the faba beans inbred lines according to their yield and quality attributes (B).

To intensely determine the multivariate relationships between the seed yield and physicochemical quality characters together with the inbred lines, HJ‐biplot analysis was carried out by comparing the eigenvalues of PC1 and PC2 of principal component analysis (PCA) for both the inbred lines and the quality traits (Fig. 2A,B). The results of the first two PC axes (PC1, 53.82% and PC2, 27.50%) accounted for about 81.78% of the total variability indicating an excellent contribution of the two PC axes to the plotted components (Fig. 2A). In the biplot, vectors of characters showing less than 90˚ angle are positively correlated, whereas those formed more than 90˚ angles are negatively correlated, and those with 90˚ angle have no correlation (Yan and Fregeau‐Reid 2008). The distance between the inbred lines is interpreted in terms of similarity. Regarding the interrelation between the parameters, the angles between ash, prolamin, carbohydrate, 100‐seed weight, and moisture content are acute indicating positive correlations among these traits. The aforementioned traits also negatively (formed obtuse and/or straight angles) associated with yield, fiber, protein, and globulin values. Despite its slightly positive correlation with globulin and moisture, the residue shows significantly negative correlation with all other parameters. Protein and fiber showed extremely positive correlation as they formed 0˚ angle between them, signifying the success of breeding for nutritionally and healthy faba bean inbred lines. Additionally, seed yield shows positive associations with fiber, protein, and the protein fractions mainly albumin, glutelin, and prolamin. These results demonstrated that improving the seed yield, which is the primary target of almost all plant breeders, would amazingly result in nutritionally rich and potentially healthy faba bean genotypes.

To assess the contribution of the inbred lines on the seed yield and quality attributes, we generated a bidimensional cluster from the mean of the yield and quality features of each inbred line (Fig. 2B). The horizontal axis groups the inbred lines based on phenotypic similarity according to their yield and seed quality characters. The differences in the color intensity indicated the values of each feature with the red color being the highest and green is the lowest. The results show three clusters of inbred lines based on their correlation with one or more of the tested parameters. The upper cluster of the inbred lines Shabah‐S5 and Seliam‐S5 shows their shared contribution to the moisture, carbohydrate, 100‐seed weight, prolamin, and ash. Interestingly, these two inbred lines show some similarities in seed shape and size (Fig. 1) and are greatly dissimilar from other inbred lines in these phenotypic characters. In addition, the big size seeds of Shabah‐S5 and Seliam‐S5 seeds may reason the high values of moisture, 100‐seed weight, and carbohydrate of these inbred lines. Strikingly, the genetic background of Shabah‐S5 and Seliam‐S5 is similar because both of them are originated from the original varieties that have been traditionally cultivated in northern Sudan. The second cluster contained the inbred lines Hudeiba/93‐5S and Ed‐damar‐S5 that shows a significant contribution to the seed yield, fiber, protein content, and quality attributes such as globulin, glutelin, and albumin. In addition, the inbred line Hudeiba/93‐S5 shows some input toward fat and ash values. The seed size of the inbred lines Hudeiba/93‐S5 and Ed‐damar‐S5 is smaller than that of Shabah‐S5 and Seliam‐S5 (Fig. 1), which might affect their marketability and consumer acceptability. Despite their little seed size, the inbred lines Hudeiba/93‐S5 and Ed‐damar‐S5 outscore Shabah‐S5 and Seliam‐S5 in terms of seed yield and nutritional quality. Thus, Hudeiba/93‐S5 and Ed‐damar‐S5 are of great importance as new faba bean inbred lines for food security in the marginal environment of subtropical areas. The last branch contains a single inbred line (Bassabier‐S5) that displays some quality performance as it shows high values of fiber, protein, globulin, and residue; however, it indicates low yield potentials. The results of biplot analysis show some dissimilarities to those of the simple correlation analysis among pairs of characters as the biplot describes the interrelationships among all characters simultaneously based on the overall contribution of the data (Yan and Fregeau‐Reid 2008). Thus, this report displays a clear picture of the associations between all the test parameters for all inbred lines concomitantly.

## Conclusion

The genetic material in the present study has sufficient variability in seed yield and its components combined with appropriate amount and variability of carbohydrate, fiber, and protein contents and quality. The inbred lines Hudeiba/93‐S5 and Ed‐damar‐S5 have high seed yield with an excellent nutritional and health qualities, and thus they are suitable candidates for cultivation in the marginal environment of the Sudan. In addition, these two inbred lines are potentially appropriate parents for faba beans breeding program to improve the faba bean seeds yield and nutritional value, and subsequently reduce the protein deficiency malnutrition in developing countries.

## Conflict of Interest

The authors declare to have no conflict of interest.
